# A Spatial and Temporal Analysis of Japanese Encephalitis in Mainland China, 1963–1975: A Period without Japanese Encephalitis Vaccination

**DOI:** 10.1371/journal.pone.0099183

**Published:** 2014-06-09

**Authors:** Xiaolong Li, Xiaoyan Gao, Zhoupeng Ren, Yuxi Cao, Jinfeng Wang, Guodong Liang

**Affiliations:** 1 State Key Laboratory for Infectious Disease Prevention and Control, National Institute for Viral Disease Control and Prevention, Chinese Center for Disease Control and Prevention, Beijing, People's Republic of China; 2 Collaborative Innovation Center for Diagnosis and Treatment of Infectious Diseases, Hangzhou, People's Republic of China; 3 State Key Laboratory of Resources and Environmental Information System, Institute of Geographic Sciences and Natural Resources Research, Chinese Academy of Sciences, Beijing, People's Republic of China; University of California Davis, United States of America

## Abstract

More than a million Japanese encephalitis (JE) cases occurred in mainland China from the 1960s to 1970s without vaccine interventions. The aim of this study is to analyze the spatial and temporal pattern of JE cases reported in mainland China from 1965 to 1973 in the absence of JE vaccination, and to discuss the impacts of climatic and geographical factors on JE during that period. Thus, the data of reported JE cases at provincial level and monthly precipitation and monthly mean temperature from 1963 to 1975 in mainland China were collected. Local Indicators of Spatial Association analysis was performed to identify spatial clusters at the province level. During that period, The epidemic peaked in 1966 and 1971 and the JE incidence reached up to 20.58/100000 and 20.92/100000, respectively. The endemic regions can be divided into three classes including high, medium, and low prevalence regions. Through spatial cluster analysis, JE epidemic hot spots were identified; most were located in the Yangtze River Plain which lies in the southeast of China. In addition, JE incidence was shown to vary among eight geomorphic units in China. Also, the JE incidence in the Loess Plateau and the North China Plain was showed to increase with the rise of temperature. Likewise, JE incidence in the Loess Plateau and the Yangtze River Plain was observed a same trend with the increase of rainfall. In conclusion, the JE cases clustered geographically during the epidemic period. Besides, the JE incidence was markedly higher on the plains than plateaus. These results may provide an insight into the epidemiological characteristics of JE in the absence of vaccine interventions and assist health authorities, both in China and potentially in Europe and Americas, in JE prevention and control strategies.

## Introduction

Japanese encephalitis (JE) is an acute infectious zoonosis disease caused by infection with Japanese encephalitis virus (JEV) through mosquito bites [1], [2], [3], [4], [5]. Infection results in damage to the central nervous system. Symptoms such as sustained high fever, disturbance of consciousness, and convulsions appear after infection with JEV, and the mortality rate can be more than 30%. Furthermore, ∼30% of the survivors may have neurological sequelae, such as severe consciousness disturbance, dementia, aphasia, and limb paralysis [2], [6], [7]. JE is an internationally recognized public health problem and has been a cause for concern globally [3]. According to a report by the World Health Organization (WHO), ∼69000 JE cases occur annually worldwide and JE is prevalent in 24 countries and territories in Asia and Oceania. Thus, ∼3000 million people live in the JE-endemic area and are at risk of JEV infection [2], [3], [8].

JE is a vaccine-preventable disease, and immunization programs can prevent JEV infection [2]. Historically, Japan, Korea, and China had high incidence of JE. However, with the implementation of JE vaccination programs in the 1960s, both Japan and Korea have almost achieved a state of JE “elimination”[8]. China began implementing a JE vaccination program in the 1980s, and the incidence of JE has since been substantially reduced [9], [10]. After 2000, with the help of the WHO and Program for Appropriate Technology in Health (PATH), as well as other international organizations, other JE endemic counties in Asia have begun to implement JE vaccination programs [8]. On the other hand, as a natural focal disease transmitted by mosquitoes, the incidence of JE can be affected by several climatic factors, such as temperature and rainfall [11], [12], [13], [14], [15]. The research conducted in Malaysia confirmed that the effects of climatic factors on JE were masked by vaccination programs and cannot be estimated precisely [16]. Therefore, the JE data derived from the period without JE vaccination are of great significance to reveal the authentic epidemiology of JE and influences of climatic factors on JE incidence.

China is a highly endemic area of JE. In addition, there was a nationwide JE epidemic in China during the 1960s and 1970s, and tens of thousands cases were reported, with an incidence of over 15/100000 per year in this era of no JE vaccine intervention [9], [10]. As China was experiencing the “Cultural Revolution” at that time, the functions of government were weakened and the public health authorities almost failed to implement any interventions. Thus, China experienced a natural JE epidemic period with no interventions from the 1960s to 1970s. Then, there comes the question that what the epidemiology of JE was in the absence of vaccine interventions during that period in China. In this study, we examined the spatial and temporal distribution of JE cases during the period 1963 to 1975 in mainland China. By combining with meteorological data, the effects of rainfall and temperature on JE in several geomorphic units are also discussed. The results not only provide data supporting the prevention and control of JE in endemic regions that have not widely implemented JE vaccination programs, but are also of practical significance for prevention and control of JE in potentially endemic regions.

## Materials and Methods

### Data collection and management

Since 1950, JE has been included in the national notifiable infectious diseases list [9]. The data on reported cases and annual incidence of JE from 1963 to 1975 were obtained from the China Information System for Disease Control and Prevention (CISDCP) [17], and covered the whole nation, including 29 provinces, municipalities, and autonomous regions (the national administrative divisions consisted of 29 provinces, municipalities, and autonomous regions during this period), among which Xinjiang, Tibet, and Qinghai had no reported cases during the study period and were classified as JE-free areas.

The meteorological data, including the monthly precipitation and monthly mean temperature, from 1963 to 1975 were obtained from the China Meteorological Data Sharing Service System in a grid format [18]. Due to the obvious seasonal distribution of JE cases, which clustered in June, July, and August in China, the temperature and rainfall in summer are more sensitive than the annual values in terms of evaluating the effects on JE. In this study, the average monthly rainfall and temperature in June, July, and August were used to represent summer rainfall and summer temperature, respectively.

### Division of geomorphic units

**Figure 1:**
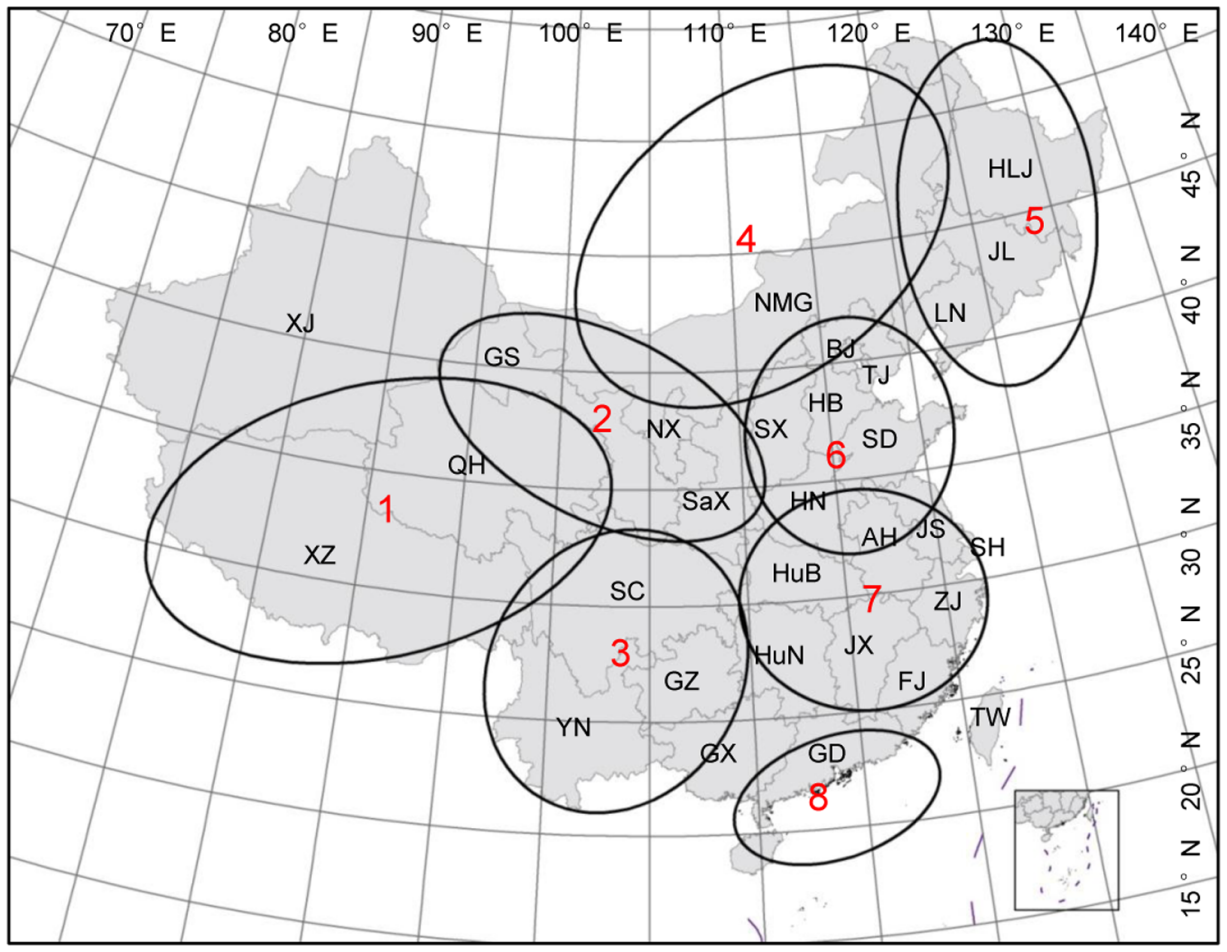
Division of geomorphic units included in this study. Region 1 represents the Qinghai-Tibet Plateau (QTP); Region 2 represents the Loess Plateau (LP); Region 3 represents the Yunnan-Guizhou Plateau (YGP); Region 4 represents the Inner Mongolian Plateau (IMP); Region 5 represents the Northeast China Plain (NECP); Region 6 represents the North China Plain (NCP); Region 7 represents the Yangtze River Plain (YRP); Region 8 represents the Pearl River Delta Plain (PRDP).

To further analyze the effects of climatic factors in different geographical environments on the incidence of JE, China was divided into eight geomorphic units and the boundaries of each geomorphic unit are represented with the boundaries of the major provincial administrative regions included (Figure 1). These geomorphic units were as follows: the Qinghai-Tibet Plateau (QTP), which consists of Qinghai and Tibet; the Loess Plateau (LP) consisting of Shaanxi, Gansu, and Ningxia; the Inner Mongolian Plateau (IMP), which consists of the Inner Mongolian Autonomous Region; the Yunnan-Guizhou Plateau (YGP) consisting of Yunnan, Guizhou, and Sichuan; the Northeast China Plain (NECP), which consists of Heilongjiang, Jilin, and Liaoning; the North China Plain (NCP) covering Hebei, Beijing, Tianjin, Shanxi, Shandong, and Henan; the Yangtze River Plain (YRP), which consists of Hunan, Hubei, Anhui, Jiangsu, Jiangxi, Zhejiang, and Shanghai; and the Pearl River Delta Plain (PRDP), which mainly includes Guangdong province. The annual incidence of JE in each plateau or plain was calculated from the sum of reported JE cases within the regions divided by the population size in the corresponding year. In addition, the summer temperature and summer rainfall for each region were derived from the gridded meteorological datasets using the zonal statistics to summarize the values within each region. Zonal statistical analyses were conducted using the Spatial Analyst module in the ArcGIS software (version 9.3; ESRI, Redlands, CA).

### Cluster analysis

The Local Indicators of Spatial Association (LISA) was used to describe the spatial clusters of JE incidence at the provincial level between 1963 and 1975. The spatial correlations or spatial clusters between the value of a given location and the average of neighboring values in the surrounding locations were represented on the LISA cluster maps by calculating the Local Moran's *I*, which ranged from −1 to 1[11], [17], [19]. In addition, the Z-score was used to measure the significance of the spatial correlations indicated by Local Moran's *I*. A high positive Z-score indicates that the surrounding features have either similar high values (High-High) or similar low values (Low-Low), while a low negative Z-score indicates a significant (*P*<0.05) spatial outlier (High-Low or Low-High) [20]. The spatial statistics module in ArcGIS software (version 9.3; ESRI) was applied to perform LISA analysis at the provincial level to identify the cluster pattern during the JE epidemic period.

## Results

### Japanese encephalitis in mainland China in 1963–1975

**Figure 2:**
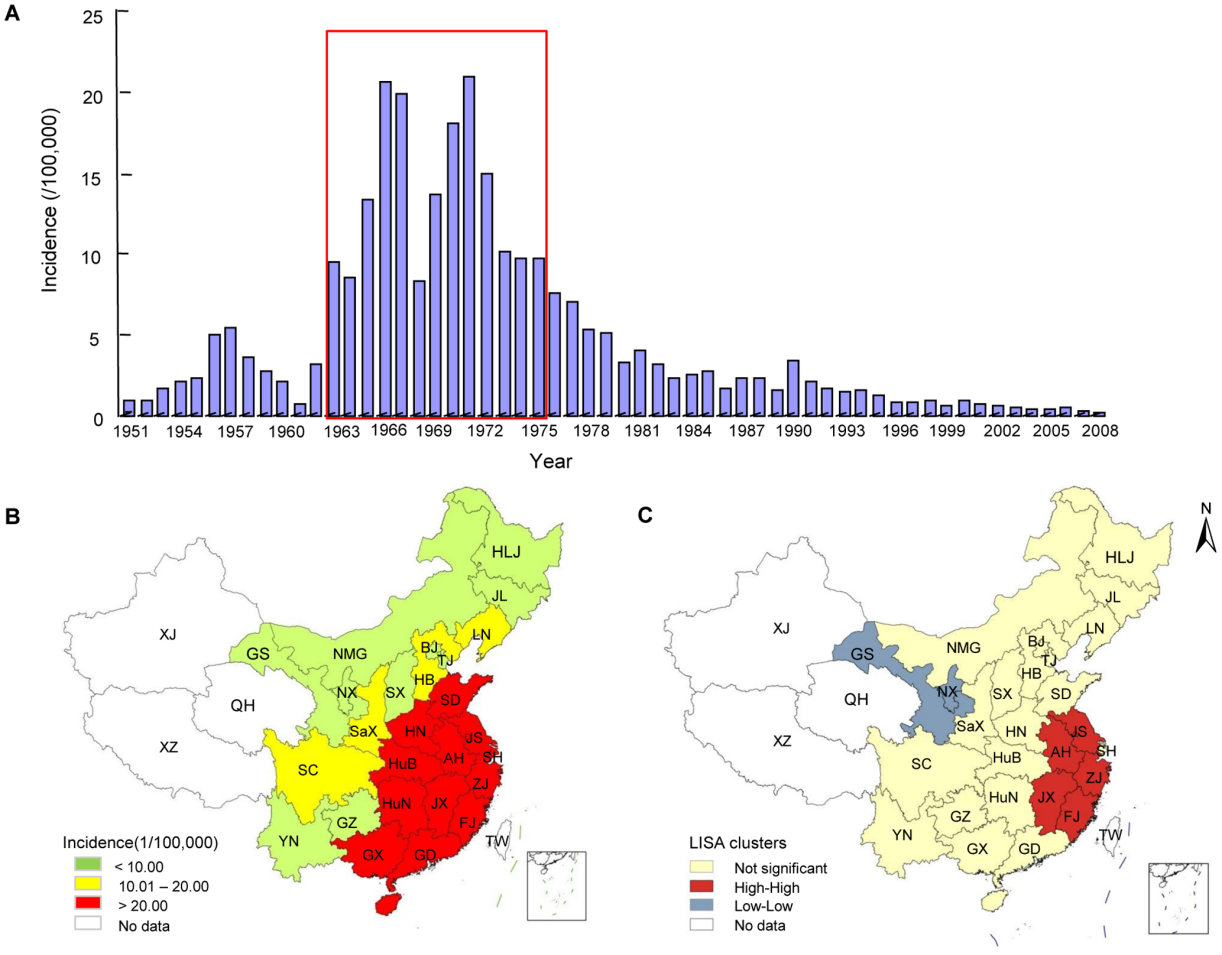
Japanese encephalitis in mainland China during 1963–1975. (A) China experienced a natural JE epidemic period with no vaccine interventions from 1963 to 1975. The bar graph of JE incidence in mainland China from 1951 to 2008 is cited from reference 10. (B) During the JE epidemic in 1971, JE incidence in 11 provinces distributed in the coastal areas of eastern China (red color) was higher than the national average (20.92/100,000). (C) LISA cluster map for JE incidence during 1963–1975 shows the center of cluster in color. High-High indicates a significant (*P*<0.05) spatial cluster of high JE incidence values; Low-Low represents a spatial cluster of low JE incidence values. **Symbols**: HLJ: Heilongjiang; JL: Jilin; LN: Liaoning; NMG: Inner Mongolia; BJ: Beijing; TJ: Tianjin; HB: Hebei: HN: Henan; SD: Shandong; SX: Shanxi; SaX: Shaanxi; NX: Ningxia; GS: Gansu; QH: Qinghai; XJ: Xinjiang; XZ: Tibet; SC: Sichuan; YN: Yunnan; GZ: Guizhou; GX: Guangxi; GD: Guangdong; GX: Guangxi; HuN: Hunan; HuB: Hubei; JX: Jiangxi; JS: Jiangsu; ZJ: Zhejiang; AH: Anhui; FJ: Fujian; SH: Shanghai; TW: Taiwan.

There were more than 1.4 million reported JE cases in China from 1963 to 1975, accounting for almost 60% of the total reported JE cases since 1950. With the exception of Xinjiang, Tibet, and Qinghai, all provinces, municipalities, and autonomous regions in China have reported cases of JE. In addition, the incidence of JE during this period was between 8.32/100000 and 20.92/100000 (Figure 2A). The epidemic peaked in 1966 and 1971, with about 150000 and 170000 cases of JE reported, representing annual JE incidence rates of 20.58/100000 and 20.92/100000, respectively.

The spatial distribution of JE around the country in 1971 is shown in Figure 2B. The incidence of JE in each province was between 1.59/100000 and 53.06/100000, and there were 10 provinces with JE incidence higher than the national average (20.92/100000). Figure 2B shows that the endemic regions can be divided into three classes including high, medium, and low prevalence regions, according to the prevalence rates. The high-prevalence regions, including Shandong, Henan, Anhui, Hunan, Hubei, Jiangsu, Jiangxi, Zhejiang, Shanghai, Fujian, Guangdong, and Guangxi, have JE incidence greater than 20/100000, and these 12 provinces are distributed in mainly the North China Plain, the Yangtze River Plain, and the Pearl River Delta Plain. Regions with medium JE incidence of between 10/100000 and 20/100000 include Liaoning, Hebei, Shaanxi, and Sichuan, which are located in central and southwestern China. The low-prevalence regions with incidence less than 10/100000 consist of 10 provinces; i.e., Heilongjiang, Liaoning, Inner Mongolia, Beijing, Tianjin, Shanxi, Ningxia, Gansu, Yunnan, and Guizhou, which are located mostly in the Northeast China Plain, the Inner Mongolian Plateau, the Loess Plateau, and the Yunnan-Guizhou Plateau.

### Cluster analysis

The results of LISA analysis indicated that the hot spots (High-High) were concentrated mainly in Jiangsu, Zhejiang, Anhui, Hunan, and Fujian provinces, which covered most of the Yangtze River Plain geographically (Figure 2C). The average JE incidence during the period from 1963 to 1975 in the provinces included in the hot spot were between 15/100000 and 22/100000 (Table 1). Moreover, the JE incidence in these provinces in 1971 were higher than 20/100000. Ningxia and Gansu, located in the Loess Plateau in northwestern China, were identified as cold spots (Low-Low) (Figure 2C); the JE incidence in these two provinces remained relatively low (around 1/100000) between 1963 and 1975 (Table 1).

**Table 1:** Japanese encephalitis in different geomorphic units, 1963–1975.

Geomorphic unit		Number	Altitude (m)	Longitude	Latitude	Provinces included	JE case NO.	Average incidence (1/100000)
Plateau	QTP	1	4000–5000	73°–104°E	26°–39°N			
						Qinghai	0	0
						Tibet	0	0
						sub-total	0	0
	LP	2	1500–2000	103°–114°E	34°–40°N			
						Shaanxi	29071	9.29
						Gansu	1584	0.75
						Ningxia	411	1.35
						sub-total	31243(2.53%)	5.62
	YGP	3	1000–2000	100°–110°E	23°–27°N			
						Yunnan	13520	4.09
						Guizhou	13218	4.54
						Sichuan	97438	9.08
						sub-total	124176(10.08%)	4.31
	IMP	4	1000–1400	106°–121°E	40°–50°N			
						Inner Mongolia	177(0.001%)	0.22
Plain	NECP	5	<200	118°–128°E	40°–48°N			
						Heilongjiang	584	0.19
						Jilin	12467	4.43
						Liaoning	51154	12.57
						sub-total	64205(5.21%)	6.26
	NCP	6	<50	114°–121°E	32°–40°N			
						Hebei	62152	10.25
						Beijing	15675	15.62
						Tianjin	5186	8.71
						Shanxi	7841	2.93
						Shandong	151403	19.08
						Henan	149110	19.65
						sub-total	391367(31.77%)	15.21
	YRP	7	<50	110°–120°E	28°–33°N			
						Anhui	92088	18.39
						Hunan	83774	14.74
						Hubei	81220	15.98
						Jiangsu	145969	22.14
						Jiangxi	65111	19.98
						Zhejiang	84649	20.82
						Shanghai	21207	15.01
						sub-total	574018(44.6%)	18.56
	PRDP	8	<50	112°–115°E	21°–24°N			
						Guangdong	110737(8.99%)	20.23
Total							1231718(100%)	

QTP: the Qinghai-Tibet Plateau; LP: the Loess Plateau; YGP: the Yunnan-Guizhou Plateau; IMP: the Inner Mongolian Plateau; NECP: the Northeast China Plain; NCP: the North China Plain; YRP: the Yangtze River Plain; PRDP: the Pearl River Delta Plain.

### Japanese encephalitis in different geomorphic units

Cluster analysis demonstrated differences in JE incidence among geographical environments. Among the eight geomorphic units included in this study (Figure 1), the plateaus range from 73° to 121° east longitude and 23° to 50° north latitude, with average altitudes of 1000–2000 m (Table 1), with the exception of the Qinghai-Tibet Plateau (average altitude, 4000–5000 m); the plains range from 110° to 128° east longitude and 21° to 48° north latitude, with an average altitude of less than 200 m.

Thus, the differences in JE incidence among the eight geomorphic units during the epidemic period were determined (Table 1). There were a total of 1.2 million reported JE cases in these geomorphic units from 1963 to 1975, most of which (85.36%) were concentrated in regions with an average altitude of less than 50 m. The average annual JE incidence in these regions was between 10/100000 and 25/100000. For example, Jiangsu province, located in the Yangtze River Plain, had an average altitude of less than 50 m, while the average JE incidence between 1963 and 1975 was 22.14/100000 (Table 1). In contrast, regions with average altitudes of 1000–2000 m had fewer JE cases and lower JE incidence. For example, the average JE incidence between 1963 and 1975 in Yunnan and Guizhou, located in the Yunnan-Guizhou Plateau, was 4.09/100000 and 4.54/100000, respectively (Table 1). Moreover, there were no reported JE cases in regions with average altitudes of greater than 2000 m, including Qinghai and Tibet. From the perspective of geographic coordinates, the JE cases were distributed mostly between 21°–40° north latitude and 110°–121° east longitude (Table 1, Figure 1). In addition, the northern regions of 40° north latitude had lower JE incidence rates and the western regions of 100° east longitude had no reported JE cases.

**Figure 3:**
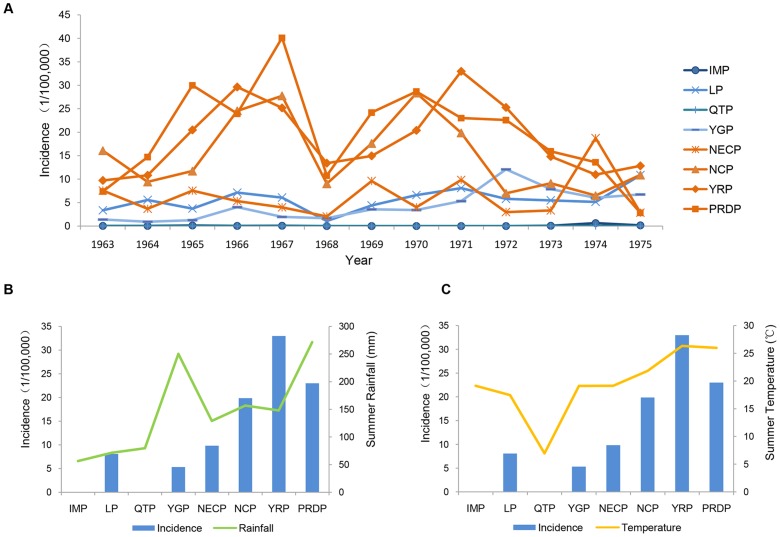
Climatic factors and Japanese encephalitis in different geomorphic units. (A) Comparison of the JE incidence in different geomorphic units shows that JE incidence in the plains was higher than that in the plateaus during 1963–1975. (B) The differences among eight geomorphic units in JE incidence and summer rainfall in 1971. There were no JE reported cases in the QTP and the IMP in 1971. Due to the affection of the southwest monsoon in summer, the YGP shows abundant summer rainfall. (C) The differences among eight geomorphic units in JE incidence and summer temperature in 1971. The summer temperature in the QTP was below 10°C in 1971 because of its high altitude (4000–5000 m).

The numbers of reported JE cases in Table 1 indicated that the JE epidemic area was distributed mainly in the plains, where the reported cases accounted for 90.57% of the total. Among these plains, the Yangtze River Plain and the North China Plain had the most JE cases, which corresponded to the results of cluster analysis during the natural JE epidemic period (Figure 2C). The JE incidence in the plains was markedly higher than those in the plateaus (Figure 3A). With regard to JE incidence in plateaus, there were no reported JE cases in the Qinghai-Tibet Plateau, and the incidence in the Inner Mongolian Plateau was consistently less than 1/100000, while those in the Loess Plateau and the Yunnan-Guizhou Plateau were less than 10/100000. With the exception of the Northeast Plain, which is located at higher latitudes (40°–50° north) and had an incidence of less than 10/100000, the JE incidence on the plains was always greater than 10/100000 (Figure 3A). The JE incidence of the North China Plain, the Yangtze River Plain, and the Pearl River Delta Plain was typically between 20/100000 and 30/100000 at the peak of the epidemic; a high incidence of 40.11/100000 occurred in Guangdong, which is located in the Pearl River Delta Plain.

### Climatic factors and Japanese encephalitis

Generally, more cases of JE occurred in geomorphic units with more abundant summer rainfall and higher summer temperature, and vice versa. In 1971, for example, when the epidemic peaked, geomorphic units with more abundant summer rainfall had a higher incidence of JE (Figure 3B); the summer rainfall in 1971 in the Pearl River Delta Plain was 270 mm and the JE incidence was 23/100000. In contrast, geomorphic units with less summer rainfall, such as the Loess Plateau, where the summer rainfall in 1971 was 70 mm, had a lower incidence of JE; the average incidence of JE in the Loess Plateau in 1971 was 8/100000.

The plateaus were relatively dry and the rainfall in summer usually did not exceed 100 mm (Figure 3B). As an exception, the Yunnan-Guizhou Plateau shows abundant rainfall because of its location in a subtropical humid area that is affected by the southwest monsoon in summer; the summer rainfall in this area reached 250 mm in 1971. The incidence of JE in plateaus was less than 10/100000, and no cases of JE were reported in the Qinghai-Tibet Plateau or the Inner Mongolia Plateau. In contrast, the plains were humid with abundant rainfall in summer; the summer rainfall in the Northeast China Plain, the North China Plain, and the Yangtze River Plain was 100–150 mm, and the summer rainfall in the Pearl River Delta Plain reached 270 mm due to the effects of the East Asia Monsoon. Accordingly, the incidence of JE in plains was greater than 20/100000, which was significantly higher than that of the plateaus (Figure 3B). Moreover, the incidence of JE in the Yangtze River Plain was greater than 30/100000. Similar to 1971, the other years during the natural epidemic period showed identical features; i.e., more abundant summer rainfall was correlated with a higher incidence of JE.

Figure 3C shows a comparison of the summer temperature and incidence of JE in various geomorphic units in 1971. The results indicated that geomorphic units with higher summer temperatures had higher incidence of JE, and vice versa. For example, the summer temperatures in all of the plateaus in 1971 were below 20°C, while the incidence of JE was all less than 10/100000; especially, in the Qinghai-Tibet Plain with an average altitude between 4000 m and 5000 m, the summer temperature was only 7°C in 1971, and there were no reported cases of JE. However, the summer temperature in the plains ranged between 20°C and 30°C. The summer temperatures in both the Yangtze River Plain and the Pearl River Delta Plain were around 26°C, and the incidence of JE in these two plains was 32.97/100000 and 23/100000, respectively.

**Figure 4:**
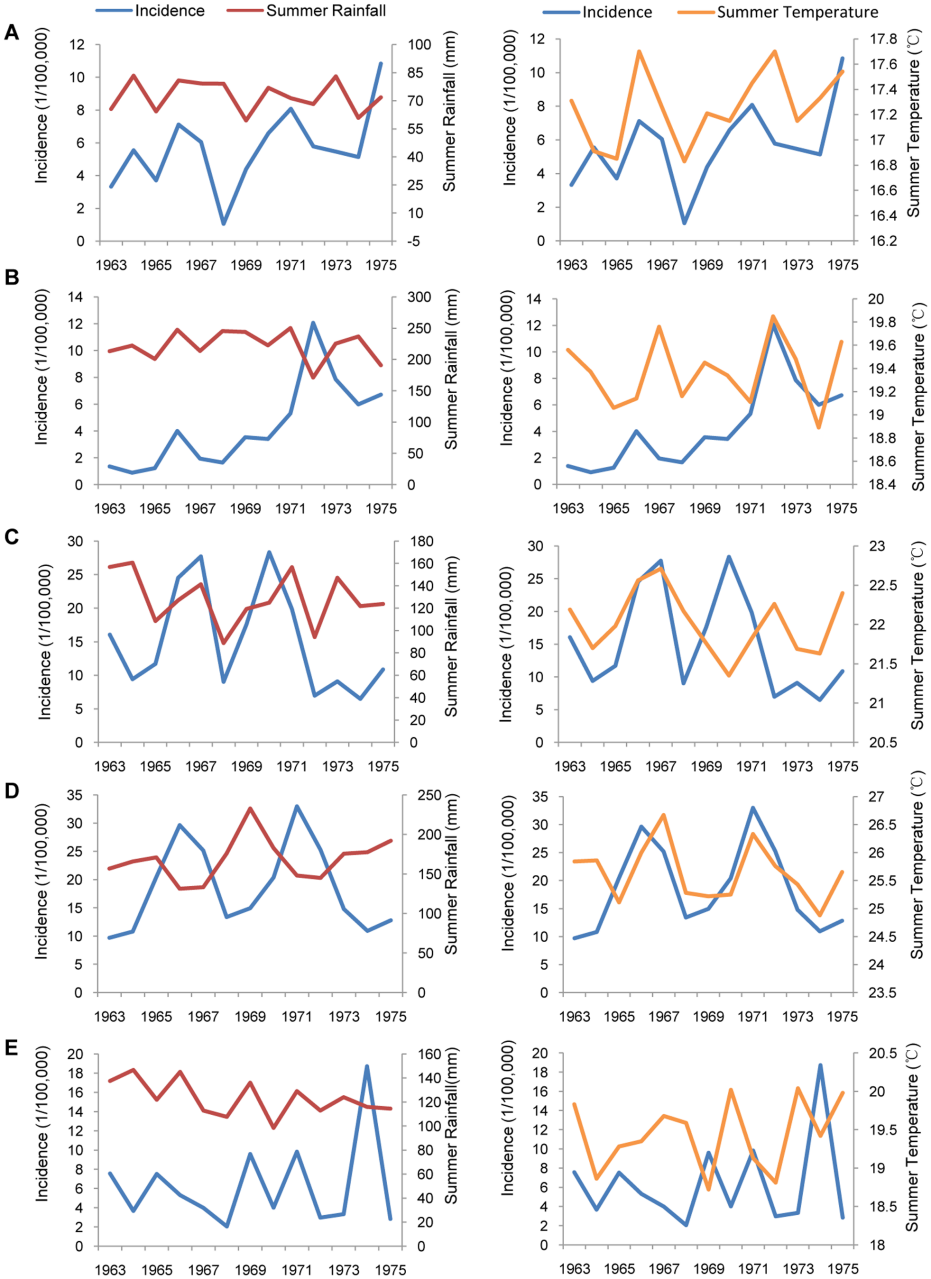
Correlations between climatic factors and Japanese encephalitis in different geomorphic units. The correlations of summer rainfall and summer temperature with JE incidence varied among different geomorphic units. A) the Loess Plateau; B) the Yunnan-Guizhou Plateau; C) the North China Plain; D) the Yangtze River Plain; E) the Northeast China Plain.

Furthermore, we illustrated the relations between climatic factors, such as summer rainfall and summer temperature, and the incidence of JE inside each geomorphic unit between 1963 and 1975 (Figure 4). For example, in the Loess Plateau, increasing summer rainfall was consistently correlated with an upward tendency of JE incidence during the epidemic period. Higher summer temperature was associated with a higher JE incidence (Figure 4A). The JE incidence and summer rainfall in the Yunnan-Guizhou Plateau showed the opposite tendency, while JE incidence and summer temperature showed the same tendency after 1970 (Figure 4B). The North China Plain showed the same trend between JE incidence and summer rainfall, while there was no obvious relationship between JE incidence and summer temperature (Figure 4C). In contrast to the North China Plain, the Yangtze River Plain showed a consistent tendency between the incidence of JE and summer temperature, while there was no obvious relationship between JE incidence and summer rainfall (Figure 4D). Due to the low JE incidence in the Northeast China Plain before 1973, there were no obvious similarities or differences in the relationships between JE incidence and summer rainfall or summer temperature (Figure 4E).

## Discussion

Without vaccination programs, the JE epidemics that occurred in the 1960s and 1970s in mainland China placed a huge burden on society. The large scale of the outbreaks and the high incidence have made this viral encephalitis pandemic almost unique not only in Asia but also globally [9], [10].

It is known that JEV is transmitted in an enzootic cycle involving mosquitoes, birds and pigs. Culex species, especially *Culex tritaeniorhynchus* which breed in rice paddies, are the major vector of JEV in Asia. Pigs act as the amplifying hosts, increasing human risk of exposure to JEV especially in some parts of Asia where pigs are kept near homes [4]. Hence, mosquito density, pig farming, area of rice paddies and the distance between residents and pigsties or rice paddies are the main natural and social factors affecting the spread of JEV in local areas. In this study, LISA analysis detected the reported JE cases during the JE epidemic from 1963 to 1975 mainly clustered in the Yangtze River Plain (region 7 in Figure 2), and ∼85% of JE cases occurred in the plains (regions 6, 7, and 8 in Figure 2) with low altitudes (<50 m). The incidence in these regions was markedly higher than those in the plateaus (regions 1, 2, 3, and 4 in Figure 2). For the plains with low altitudes, the summer rainfall there is abundant and the summer temperature is high. In addition with the high population density and the vast area of intensive pig farming and rice paddies which provide a suitable habitat for mosquitoes, the reproduction and propagation of JEV would be increased and JE epidemics are more likely to occur in these regions [9], [21]. While, due to the higher altitude, lower temperature and rainfall, lower population density, and the livestock-based agriculture with relatively sparse pigsties and rice paddies in plateau areas, the breeding of mosquitoes and the circulation of JEV would be hindered [21], and fewer JE cases occurred in these regions during the JE epidemic period. As for the plains located at high latitude, such as the Northeast China Plain located between 40° and 50° north latitude, the high latitude led to a lower annual mean temperature in these plain regions, and the JE incidence remained low as well. Furthermore, the Qinghai-Tibet Plateau (located between 25°and 50° north latitude, 80° and 100° east longitude) had an average altitude of 4000–5000 m and temperatures lower than 10°C in summer; there were no JE cases in these areas [9].

The temperature and rainfall are considered to be the two major climatic factors affecting the replication and propagation of JEV, by influencing the life cycle and population density of mosquitoes [11], [22], [23], [24], or by directly impacting the reproduction rate of JEV in mosquitoes [15]. Increased temperature and rainfall can reduce the maturation time of the mosquito larvae and rapidly increase the population density of mosquitoes [25], which would increase the chance of mosquito bites and JEV infection. These factors can in part explain why the high JE incidence in plains was associated with abundant summer rainfall and high summer temperature. However, it was observed that JE incidence and summer rainfall in the Yunnan-Guizhou Plateau showed the opposite tendency in Figure 4B. These observations might be directly related to the special geographic features of the Yunnan-Guizhou Plateau. The level of summer rainfall in this region is relatively high compared to the other regions; however, because of the typical mountainous characteristics of this plateau, rainwater does not gather readily, and currents can destroy mosquito-breeding habitats and the mosquito population density could be reduced. Therefore, the incidence of JE may not go up with increases in rainfall in this region.

In common with other natural focal diseases, the range of JE-endemic areas has shown a propensity to expand to some new areas [26], [27]. In 1995, a widespread JE outbreak firstly emerged on the Torres Strait islands of northern Australia [28]. Due to the low altitude and tropical climate, the northern Australia has high temperature and abundant rainfall in summer, which is conducive to mosquito breeding. In addition, suitable vector mosquitoes (*Culex annulirostris*) and vertebrate hosts (pigs) existing in northern Australia provided the enzootic cycles for the dispersal of JEV. Soon afterward, JE reemerged in this region and gradually spread to the mainland Australia in 1998 [29]. Similar with Australia, Tibet in China is another example. No JE cases were reported in Tibet since 1950, where the average altitude is 4000 m. However, numerous *Culex tritaeniorhynchus* were collected in Tibet in 2009, and the JEV was isolated from these mosquito samples. Moreover, 33.3% (22/66) of pig sera were JEV IgM antibody-positive and 27.4% (68/248) of human serum samples were positive for neutralizing antibody in local, indicating that Tibet, a traditionally JE-free region, has become a natural epidemic focus of JEV [30].

In addition, based on the results of this study, the potentials that further dispersal of JEV to traditional non-JE regions were discussed. For example, JEV could be transmitted to the south of Europe by migrating birds and cause epidemics. The reason is that the high summer temperature and appropriate rainfall conditions in these regions are suitable for breeding of mosquitoes and there are large numbers of suitable vectors for JEV, such as *Culex pipiens* and *Culex quinquefasciatus* [31], as well as pigs to act as hosts [32]. Similar predictions were made in previous studies [33], [34]. However, it is unlikely that JEV could be transmitted to the east of Russia because of the cold weather and a limited range of mosquito species [35].

These data collected from the disease reporting system provided a foundation for the present study. Based on these data, the spatial and temporal pattern of JE epidemic occurred during the period without vaccination was examined in this study. Further, we discussed the impacts of climatic and geographic factors such as temperature, rainfall, and elevation on JE epidemic as well. In conclusion, JEV has shown a propensity to circulate and cause epidemic in plains with low altitude, relatively high temperature and abundant rainfall, and intensive pig farms. Therefore, for some new regions with similar natural and social environments for JEV transmission, such as Europe and Americas, systematic surveillance works on mosquito species and density, and JEV seroprevalence in pigs are needed in order to detect the JE cases timely and make the early warning for the introduction of JEV into local areas.

Also, this study had several limitations due to the use of historical data. During the study period, 1963–1975, there were no JEV-specific detection methods and techniques such as enzyme-linked immunosorbent assay (ELISA) and immunofluorescence assay (IFA) applied in China. Therefore, the diagnosis of JE cases was generally based on the epidemiological history and clinical symptoms, lacking of the confirmation from specific laboratory tests. In addition, the data regarding JE epidemics were limited and only the number and incidence of JE cases for each province per year during the 1960s and 1970s were accessible; demographic data (such as gender, age, occupation), JE case data in county level and monthly statistical data were lacking. Therefore, we analyzed the spatial cluster of JE cases from 1963 to 1975 on the basis of only the number of JE cases and the incidence at the provincial level per year, which may lead to ecological fallacy to some extent [36], [37]. Meanwhile, a more in-depth analysis to evaluate the effects of climatic factors like rainfall and temperature on JE by using statistical models was not possible due to lacking of more detailed data such as county-level JE data and meteorological data, which is regrettable for this study.
